# The State-of-the-Art of Sensors and Environmental Monitoring Technologies in Buildings

**DOI:** 10.3390/s19173648

**Published:** 2019-08-22

**Authors:** Hasan Hayat, Thomas Griffiths, Desmond Brennan, Richard P. Lewis, Michael Barclay, Chris Weirman, Bruce Philip, Justin R. Searle

**Affiliations:** SPECIFIC, College of Engineering, Swansea University, Wales SA1 8EN, UK

**Keywords:** sensors, communications, selection, placement, batteries, energy harvesting, buildings

## Abstract

Building energy consumption accounts for 30%–45% of the global energy demand. With an ever-increasing world population, it has now become essential to minimize the energy consumption for the future of the environment. One of the most crucial aspects in this regard is the utilization of sensing and environmental monitoring technologies in buildings as these technologies provide stakeholders, such as owners, designers, managers, and occupants, with important information regarding the energy performance, safety and cost-effectiveness of the building. With the global sensors market value predicted to exceed $190 billion by 2021 and the number of sensors deployed worldwide forecasted to reach the ‘1 Trillion’ mark by 2025, a state-of-the-art review of various commercially-viable sensor devices and the wide range of communication technologies that complement them is highly desirable. This paper provides an insight into various sensing and environmental monitoring technologies commonly deployed in buildings by surveying different sensor technologies, wired and wireless communication technologies, and the key selection parameters and strategies for optimal sensor placement. In addition, we review the key characteristics and limitations of the most prominent battery technologies in use today, different energy harvesting sources and commercial off-the-shelf solutions, and various challenges and future perspectives associated with the application of sensing and environmental monitoring technologies within buildings.

## 1. Introduction

The demand for efficient energy sources and novel forms of energy has been a constant since ancient times and has become even more prominent now with the worldwide demand to reduce carbon emissions and fossil-fuel-based energy sources. It is reported that the UK service sector accounts for 14% of the country’s total energy consumption [1], and in this regard, the UK Government aims to reduce carbon emissions by up to 60% by the year 2050 in comparison to figures reported in 2006 [2]. At the same time, the global energy consumption is expected to increase from 575 quadrillion Btu reported in 2015 to up to 736 quadrillion Btu by the year 2040, predicting a staggering increase of up to 28% [3]. In this context, environmental monitoring and control via sensors, and communications and networking technologies designed to complement these sensors, play a significant role in improving the process of generation, storage and release of energy, minimizing the overall energy consumption, and ensuring the overall comfort and safety of occupants in today’s buildings [4].

**Figure 1:**
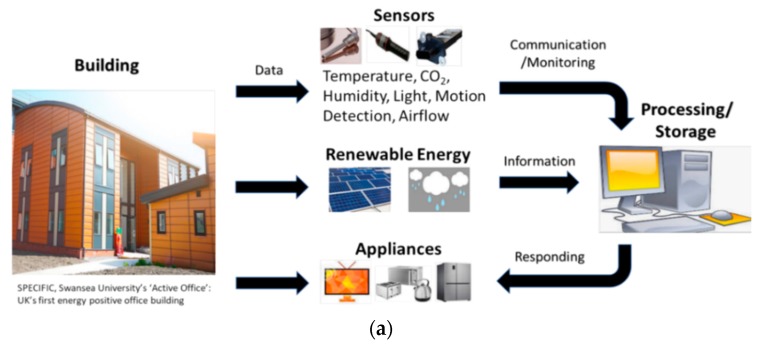

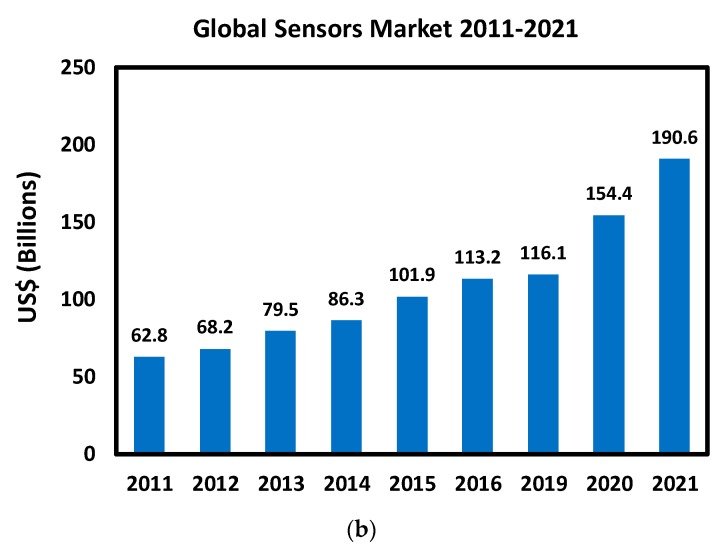
(**a**) Overview of sensing and environmental monitoring in buildings (**b**) Global sensors market growth (in billions of dollars) from 2011 to 2021 [17].

Buildings represent a significantly higher energy consumption percentage in comparison to other sectors worldwide and it is estimated that their average consumptions account for 30%–45% of the global energy demand [5]. Figures for the European Union present a similar percentage with buildings accounting for approximately 40% of the total consumption and up to 36% of greenhouse gas emissions, with commercial buildings (primarily office and educational buildings) representing the largest energy consumptions [6,7]. The primary uses of energy in such establishments include heating, cooling, lighting and the powering of electronic appliances, and as of 2011, approximately 81% of the energy produced for these necessities comes from the burning of fossil fuels [8] (attributed as one of the leading causes of global climate change). As a result, the building sector has been one of the main focuses of government initiatives in recent years to monitor and reduce energy consumption and continues to be the center of attention for academic and industrial research activities worldwide, e.g., GAIA [9], OrbEEt [10], TRIBE [11] and SPECIFIC [12]. Although research in recent years indicates that modern-day buildings have huge potential for reductions in energy consumption and carbon emissions, (e.g., Chung et al. [13] determined that the overall energy consumption in a modern-day academic building can be reduced by between 6%–29%, and the Carbon Trust reported that carbon reductions of up to 70%–75% can be achieved in non-domestic buildings [14]), large inconsistencies have also been observed in such predictions, i.e., electricity demands in academic buildings and general offices can be up to 60%–85% higher and energy consumptions of non-domestic buildings can be up to twice as much as predicted in some cases [15,16]. Therefore, it is essential that modern-day buildings are equipped with accurate and efficient sensing and communication technologies as these technologies provide stakeholders, such as building designers, owners, managers and occupants, with important information regarding the performance, safety and cost-effectiveness of the building, and enable the minimization of inconsistencies in future energy demand predictions (see Figure 1a).

The global sensors market has seen extraordinary growth since 2011, which can be fathomed by the fact that sensors accounted for a worldwide market valued at more than $100 billion in 2015 (rising from $62 billion in 2011 at a compound annual rate of 12.6%, see Figure 1b). This figure is expected to exceed $154 billion by 2020, with environmental sensors accounting for more than 13% (i.e., $20.5 billion) of this amount [17]. At the same time, the forecast date for hitting ‘1 Trillion’ sensors worldwide has already been brought forward from 2030 to 2025, adding further strength to these predictions [18]. In this regard, there are a multitude of sensor technologies currently available on the market for environmental monitoring in buildings with varying levels of complexities and functionalities, such as temperature, carbon dioxide, humidity, occupancy, light, and airflow sensors. To complement these sensors, a plethora of communication and networking technologies, such as Ethernet, Power Line Carrier, Zigbee, Bluetooth, Wifi, EnOcean, BACnet, Modbus, 6LoWPAN, Z-Wave and LoRaWAN are also commercially available today. These communication technologies are essential for the reliable and secure communication and storage of high volumes of data acquired from sensors, and are expected to play an even more significant role in the integration of smart buildings and grids of the future [19].

In the following sections, we present a detailed review of various sensors and environmental monitoring technologies commercially available and widely used today for building applications, and in doing so, we aim to answer the following key questions:

(i) What is the current state-of-the art of commercially available sensors and monitoring devices in terms of operation principles, accuracy, measurement range, response times, portability, power consumption, and cost? (Section 2)
(ii) What is the current state-of-the-art of commercially available wired and wireless communication technologies in terms of operation principles, connectivity and scalability, measurement range, data rates, power consumption and market adoption? (Section 3)
(iii) What are the key characteristics, limitations and potential of various battery and energy harvesting technologies for sensing and monitoring applications in buildings? (Section 4)
(iv) What are the key aspects regarding the application, challenges and future perspectives of sensing and monitoring technologies in buildings? (Section 5)

## 2. Sensors and Monitoring Technologies

**Figure 2:**
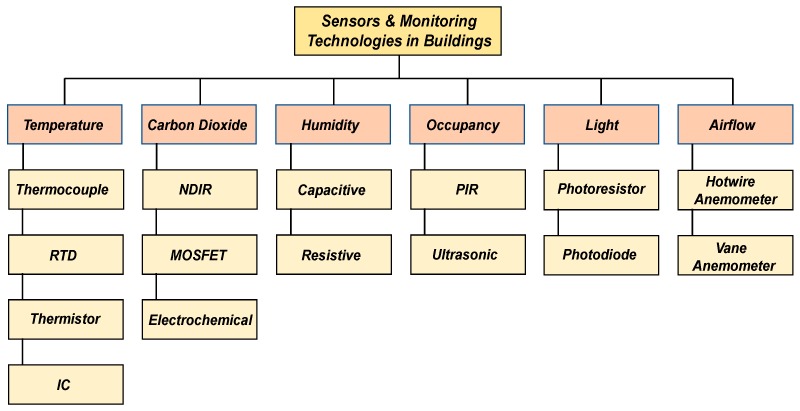
Prominent sensors and monitoring technologies for buildings applications.

Modern-day buildings can be highly adaptable to frequent changes in environmental conditions owing to a broad spectrum of semi-automated and manual systems consisting of various mechanical, electrical and electronic, thermal, and software sub-systems. Recent research studies have proposed the development and application of control algorithms [20], feedback mechanisms [21] and heating, ventilating and air conditioning (HVAC) systems [22] for building applications, and at the core of these systems and approaches are sensors and monitoring devices which feed information to control algorithms and models regarding various building parameters and its occupants. Most sensor devices available today can be used in conjunction with widely adopted controllers (e.g., PID (programmable–integrative–derivative), ON/OFF, and Fuzzy Logic controllers) and can generally be classified into two main types based on their operating principles, i.e., mechanical and electronic. Mechanical sensors are typically employed for direct control/visualization purposes and tend to be low cost, whereas electronic sensors have the added capability to convert a detected parameter into a digital value that can be used for monitoring and control, and generally tend to be more robust and scalable. In terms of communication and storage, most sensors available today can be classified into three main types, i.e., wired, wireless and portable, and in terms of applications, they are typically acquired for direct visualization, and in Building Management Systems (BMS) and HVAC systems within buildings. The following sub-sections provide a more detailed insight into various essential sensing and monitoring technologies commercially available today for use in buildings (see Figure 2) with their key characteristics summarized in Table 1 and Table 2.

### 2.1. Temperature Sensors

Temperature sensors are devices designed to measure the temperature of a flowing media such as air, water, etc., and are one of the most essential and commonly employed sensors for monitoring and control in buildings today. The four main kinds of temperature sensors currently available on the market include (1) Thermocouples; thermoelectric devices which operate based on the Seebeck effect (a phenomenon in which a voltage difference is produced between two dissimilar electrodes or semiconductor wires proportional to the temperature difference between them [23]), and are easily identified by a thermocouple probe consisting primarily of two color-coded wires, (2) Resistance temperature detectors (RTDs); devices which measure temperature based on a change in resistance in a metal resistor inside them, with Pt100 sensors (using platinum as the metal, having a resistance of 100 Ω and a typical temperature coefficient of 4 Ω/°C [24]) being the most widely adopted RTDs, (3) Thermistors; devices similar to RTDs but containing a ceramic or polymer rather than a metal inside them, and typically featuring two wires, (i.e., red for excitation and black for ground), and (4) Integrated Circuit (IC) sensors; two terminal integrated circuit temperature transducers that produce a current or voltage signal (either analogue or digital) proportional to the absolute temperature and are readily available on the market in small IC packages. Although each of the aforementioned temperature sensors will provide the end user with the required results in most cases, published literature and information available online [4,24,25,26,27,28,29,30] suggest that their respective features and properties, such as simplicity of operation, accuracy, temperature measurement range, response time, and inevitably cost can vary significantly (see Table 1).

Thermocouples are generally the most commonly used sensors in the temperature measurement world, which is attributed to their good blend of wide measurement range (−100–500 °C), fast response times (5–80 s), self-powering capability, and low cost ($6–$50). However, these devices are not as accurate (±1–4 °C) or stable (due to chemical changes such as oxidation within the sensor) as some of the other types of temperature sensors, and typically, do not possess wireless communication capabilities. RTDs, on the other hand, offer good accuracy (±0.2–1 °C), both wired and wireless capability, repeatability, higher immunity to electrical noise, and linearity (i.e., the temperature-resistance relation in RTDs is linear, whereas, in thermocouples, it tends to be an ‘S’-type plot). However, RTDs have a narrower temperature measurement range (−50–250 °C), slower response times (1–8 min), are larger in size and are relatively expensive ($30–$100). In comparison, thermistors offer exceptional accuracy (±0.05–0.5 °C), faster response times (0.2–10 s), high sensitivity, both wired and wireless capability, availability in small sizes, and a lower cost in comparison to RTDs ($20–$70). Despite these features, thermistors can only measure temperatures over a fairly limited range (−50–130 °C), are non-linear (i.e., the large non-linear effect needs to be calibrated before using it in a specified measurable range), suffer from self-heating (at higher temperatures where their resistances are lower), and can be somewhat complex to use. Finally, IC sensors are the latest of the four temperature sensors currently available and are typically available in two main types; analogue output, and digital interface devices. Although IC sensors have a narrower temperature measurement range (−40–150 °C) and moderate response times (0.5–100 s), these devices have reasonably good accuracy (±0.5–1 °C), excellent linearity, simple circuitry and small packaging (as small as 0.8 mm × 0.8 mm), low cost ($1–$15) and no need for any calibration process (as these sensors are calibrated during production testing), features which make IC sensors highly suitable for monitoring and control purposes within buildings.

### 2.2. Carbon Dioxide Sensors

Carbon Dioxide (CO_2_) is a natural component of the air surrounding us and is exhaled by human beings during respiration. The amount of CO_2_ people emit varies with activities in a quantitative manner [31]; the rate of CO_2_ an occupant generates whilst sitting stationary can be up to 0.27 L/min and this can increase up to 0.53 L/min if the same person was lifting and packing [24]. The average adult’s breath contains between 35,000 to 50,000 ppm of CO_2_, which is up to 100 times more than the outdoor air [32], and carbon emissions (both CO_2_ and carbon monoxide (CO)) can accumulate substantially depending on the number of occupants and insufficient ventilation within the building. Therefore, carbon dioxide sensors are some of the most widely used devices today as they enable the monitoring and control of carbon levels to ensure the health and safety of occupants, enhance energy efficiency, and help in maintaining Indoor Air Quality (IAQ) within buildings, while minimizing excessive air changes resulting in increased energy consumption.

CO_2_ sensors typically measure the concentration of gases in a particular space in units of ppm, and provide useful information regarding the presence, location, count and user activity within a building [33,34,35,36]. The most common type of CO_2_ sensors in this regard are NDIR (non-dispersive infrared) devices; sensors which monitor the net increase/decrease of light occurring at the wavelength at which CO_2_ absorption takes place—the light intensity is used to measure the concentration of CO_2_ in the atmosphere. NDIR sensors are widely used due to their high accuracies (±30–200 ppm), wide measuring range (0–10,000 ppm), durability and reliability. However, these sensors have relatively slow response times (30–100 s) and in some cases, have high power consumptions [37,38,39,40,41], see Table 1. In addition to CO_2_ measurements, CO and volatile organic compounds ((VOC), e.g., ethanol, formaldehyde) measurement sensors have also gained significant attention in recent years as these pollutants are also ever-present in indoor environments [42,43]. The two most commonly adopted types of CO and VOC sensors are metal oxide semiconductor (MOSFET)-based and electrochemical-based sensors. Metal oxide materials, such as tungsten oxide and titanium oxide (particularly WO_3_ and TiO_2_), tend to exhibit high sensitivities to variations in air composition [44] and, in MOSFET sensors, this mechanism is exploited to produce a change in electrical conductivity of the sensor’s material. Although MOSFET sensors are highly accurate (±30–100 ppm), have a wide measurement range (400–20,000 ppm) and possess both wired and wireless functionalities, they tend to have higher energy consumptions (as they require an electric heater to maintain the sensing element temperature within the sensor at up to 300 °C) [4], relatively slow response times (50–60 s) and short life spans. On the other hand, electrochemical sensors (devices which operate in a similar way to fuel cells and produce current outputs when exposed to air pollutants) exhibit faster response times (10–60 s), higher accuracies (±0–30 ppm) and sensitivities, longer life spans and lower energy consumptions [45], but tend to have smaller measurement ranges (0–1000 ppm) when compared with MOSFET sensors (see Table 1).

### 2.3. Humidity Sensors

Humidity—the quantification of water vapor in air—has a direct effect on the comfort of occupants, the functioning of various electrical appliances and industrial equipment, and can strongly influence various chemical and physical processes within buildings. The continuing drive to reduce energy loss from uncontrolled air infiltration by increasing the airtightness of buildings has had the unintended consequence of increasing humidity-related issues. Therefore, it has become increasingly essential to measure humidity within indoor environments, especially in control systems where the health and safety of occupants is of paramount importance.

Humidity sensors are designed to measure the amount of water vapor in air which is generally measured in terms of (1) relative humidity (measured as a function of temperature), or (2) dew point (measured as a function of pressure of the gas) or (3) absolute humidity (also known as PPM, and is independent of a temperature factor) [46,47,48]. Humidity measurement devices are generally classified into two main types: (1) capacitive and (2) resistive sensors (see Table 1). Capacitive sensors are electronic devices comprising of a dielectric material (usually a plastic or polymer) sandwiched between two metal electrodes (usually gold or platinum) and deposited on a substrate (glass, silicon or alumina). This device structure enables the formation of a ‘capacitive effect’ with the capacitance measured via the dielectric constant of the dielectric material when the humidity of the surrounding air changes. These sensors are very linear (can measure % RH (relative humidity) from 0%–100%), highly accurate (±0%–5%) and generally, have low power consumptions [48,49,50,51,52]. However, capacitive sensors require complex circuitries, regular calibration and are more expensive in comparison to other humidity sensor technologies. On the other hand, resistive sensors—electronic devices which measure electrical resistance with respect to the humidity level—tend to have a narrow measurement range (i.e., most resistive sensors are unable to measure values <5% RH) and are less accurate (±1%–10%). However, these sensors have lower costs on average, and have good response times (10–60 s), making them suitable for locations within buildings where very low measurements (<5% RH) are not necessarily required [48,49,50,51,52].

### 2.4. Occupancy Sensors

Occupancy sensors are devices used to detect occupancy within a specific space and are designed to send information to building controllers to turn/dim lights and turn equipment ON and OFF based on the presence and motion of people in a particular space. When correctly implemented, this not only helps in saving energy and provides security in buildings [53,54] but is also useful for climate control [55], making these sensors highly valuable for deployment in buildings.

Occupancy sensors can be classified into two main types based on technology: (1) Passive Infrared (PIR) and (2) Ultrasonic sensors (see Table 2). PIR sensors; devices that are able to detect movement (as the human body naturally releases infrared radiation) in a specific space using a pyro-electric element [56], are the most commonly adopted occupancy sensors for commercial and household buildings nowadays, which is attributed to their fast response times (as fast as 0.5 s), both wired and wireless capabilities, low cost ($20–$65) and passivity (i.e., these sensors do not emit any energy themselves but transmit a signal based on a change in IR radiation, instead making them very useful during lengthy idle operations when no human activity occurs). Despite these advantages, PIR sensors present some significant drawbacks, such as a requirement for direct line of sight with the object (i.e., these sensors cannot detect movement behind obstacles, e.g., walls, office cubicles, etc.), and are often unsuitable for outdoor locations due to heat interference (i.e., they are only suitable for short distance measurements (3–10 m) in indoor environments) [57]. In comparison, ultrasonic sensors; active devices which are able to transmit and receive ultrasonic sound waves based on the Doppler effect (i.e., an increase or decrease in the frequency of sound or light based on the change in distance between the source and observer), are able to measure occupancy in outdoor environments over long distances (up to 70 m distances), do not require a direct line of sight, and have fast response times (1.3 ms–30 min) [52,58,59]. Whilst these functionalities make ultrasonic sensors appealing for use in both large- and small-sized buildings, these devices come at a much higher cost to the end user ($130–$500) and can have higher power consumptions in comparison to PIR sensors due to these added capabilities. In addition, ultrasonic sensors (due to their high sensitivity) can be highly susceptible to false alarms typically triggered by activities such as movement/vibrations from a different room within the building [60,61].

In addition to PIR and Ultrasonic sensors, the application of image-based and sound-based detection systems has also been explored in recent years for occupancy measurements in buildings [62,63,64]. Image detection systems (based on video cameras being placed at locations with minimal obstructions inside and outside buildings) and sound detection systems (based on the detection of audible sound waves using microphones which convert the measured acoustic signal into an electrical signal) have the capability to provide information on occupant presence, location, count and activity. However, drawbacks such as the requirement for direct line of sight, user privacy and costly hardware in the case of image detection systems and sound waves from non-human sources triggering sensors in the case of sound detection systems can limit their large-scale deployment in buildings [57].

### 2.5. Light Sensors

Light sensors are passive devices designed to convert light energy (typically ranging in the light spectrum from ‘Infrared’ (3 GHz–400 THz) to ‘Visible’ (430–770 THz) and, in some cases, up to ‘Ultraviolet’ (800 THz–300 PHz) into an electrical output signal which can be used to switch/dim lights ON and OFF when the light level is above or below a certain limit [65].

The two most common types of daylight sensors in use today are (1) photoresistors; electronic devices whose resistance decreases with increasing light intensity and vice versa and (2) photodiodes; electronic devices that can convert light into a current or voltage signal. Both photoresistors and photodiodes generally have similar accuracies (±5%–10%), wired and wireless capabilities, and suitability for both indoor and outdoor environments. However, photodiodes have faster response times due to having both analog and digital capabilities, lower energy consumption, and tend to be cheaper in cost, which makes these devices more widely used in comparison to photoresistors for building applications (see Table 2) [50,52,66,67,68].

### 2.6. Airflow Sensors

Air velocity has a direct effect on the thermal comfort of occupants in buildings and can subsequently impact their ability to work, sleep, and carry out other daily activities [69,70]. These velocities are directly proportional to the heat exchange between occupants and the air surrounding them and are generally lower indoors (typically < 1 m/s) as compared to outdoor environments.

The most common type of sensors in use today to measure air flow inside and outside buildings are called anemometers; devices designed to measure the velocity or force of a gas in a confined flow (e.g., air flow in a duct) or unconfined flow (e.g., wind outdoors) in any particular direction, and are available in two main types, i.e., (1) hotwire anemometers and (2) vane anemometers. Hotwire anemometers are thermoelectric devices consisting of a thin wire sensing element (typically, tungsten [71] or nickel [72] due to their high material strength and high temperature coefficient of resistance) which is electrically heated to a specified temperature followed by cooling, which enables the measurement of the air velocity proportional to the cooling rate of the anemometer. Hotwire anemometers provide fast response times (0.1–5 s) but are extremely sensitive to dust, humidity, corrosion and fluctuations in temperature. As a result, these sensors have a relatively narrow measurement range (0.1–25 m/s) and are only suitable for measurements in indoor environments where air velocities tend to be low. In comparison, vane anemometers; devices that combine a wind vane (for direction of airflow) with a propeller-like device to measure air velocity and wind speed [73], have a much larger measurement range (up to 45 m/s), fast response times (up to 0.25 m/s) and rigid device structures, making them more suitable for deployment on the exterior of buildings and other harsh environments [50,52,74,75,76].

## 3. Communication Technologies and Protocols

**Figure 3:**
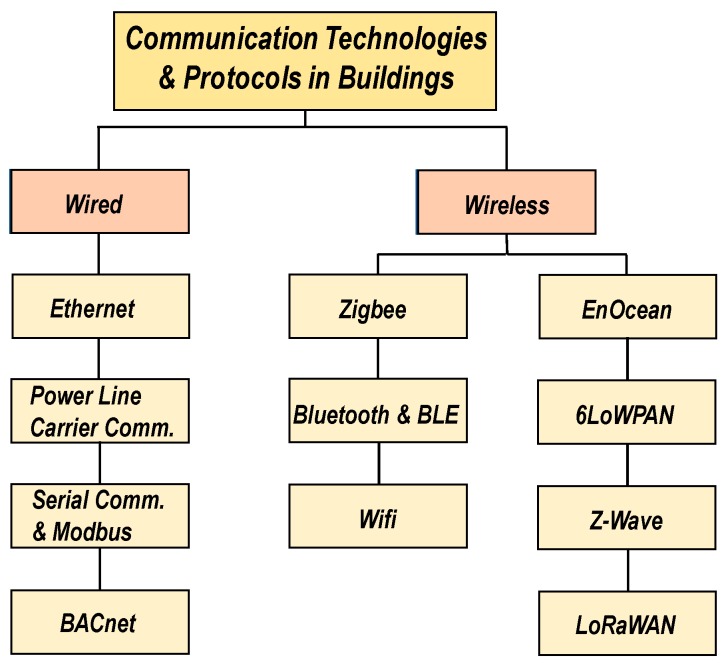
Prominent communication technologies and protocols for building applications. Ethernet, Power Line Carrier Communication (PLCC) and Serial Communications are widely adopted wired technologies whereas Zigbee, Bluetooth and Wifi are the most widely adopted wireless technologies. On the other hand, relatively new technologies and protocols, such as EnOcean, BACnet, 6LoWPAN, Z-Wave and LoRaWAN are still in their infancy in terms of market adoption.

Modern-day buildings can be equipped with a wide range of state-of-the-art wired and wireless communication technologies and protocols for the interconnection of various building systems and processes, such as the monitoring and storage of sensor information, data communication and processing in building management systems, and the functioning of equipment and utility appliances etc., (see Figure 1). Wired technologies such as Ethernet, Serial Communication (RS232/RS422/RS485), etc., tend to be more secure (as these connections are typically housed behind a Local Area Network (LAN) firewall meaning there is no transmitting data susceptible to interference), have fast data transfer speeds (as they are less affected by physical hindrances (walls, ceilings, etc.) and signal interference/distortion from other electronic devices), and tend to be more reliable in most cases (as they are less prone to disconnections, and do not require constant debugging and troubleshooting). On the other hand, wireless technologies such as Zigbee, WiFi, Bluetooth, EnOcean, etc., are more scalable and mobile (as there is no need for extensive hardware installations especially, inside walls, ceilings, etc.) and generally, cost less to the end user (as there are no additional cable installation/repair and labor costs). Although wired technologies have been more commonly used over the years, recent literature suggests that wireless technologies are now becoming increasingly ubiquitous due to vast improvements in speed, security, reliability, and an ever-increasing variety of technologies and standards to choose from [77,78,79,80,81]. Although both sets of technologies have a place in the market, it is vital to assess the viability and current status of each technology to ensure that building designers, owners and occupants can choose the most suitable technology candidate to meet their respective requirements. The following sub-sections present an overview of the most prominent and commercially available wired and wireless communication technologies and protocols used in buildings (shown in Figure 3) with their key characteristics compared in Table 3.

### 3.1. Ethernet

Ethernet (IEEE 802.3) is one of the cheapest, most widely used wired technologies for LAN and Wide Area Network (WAN) connections within buildings which is attributed to its high data rates (ranging from 10 Mbps up to 100 Gbps), distance range (up to 100 m) and high immunity to noise. Ethernet connections are typically based on one of three types of cables, i.e., coaxial, twisted pair or fiber optic, with the cable connecting to a central hub or switch in a star topology and providing the user with the possibility of different data rates, i.e., 10 Mbps, 100 Mbps, 1 Gbps, 10 Gbps and 100 Gbps. In addition, various solutions for real-time applications, (e.g., EtherCAT [82], SERCOS-III [83] and PROFINET [84]) are also presently available to the end user, further enhancing its viability. Although Ethernet provides high immunity to noise and is a fast and secure technology, a significant drawback is the difficulty in making any changes to the network once it is placed. It was also suggested in [85] that Ethernet is not best suited for real-time applications due to a lack of timeliness guarantees, operational flexibility and bandwidth efficiency. To address this limitation, the authors proposed a new protocol, i.e., FTT-Ethernet, which enables online admission control for real-time operation, and data structures and mechanisms designed to support dynamic QoS (quality-of-service) management. More recently, a new time-triggered protocol designed for decentralized control applications has also been presented in [86], which can reduce the node forwarding delay and cycle time during the communication process, with the authors claiming that the proposed protocol has a shorter cycle time when compared with other solutions such as EtherCAT and PROFINET.

### 3.2. Power Line Carrier Communication (PLCC)

Power Line Carrier Communication (PLCC) uses the mains power line, i.e., electricity grid, radio, etc., to transmit and receive data, typically by injecting a high-frequency carrier onto the AC power line and modulating the carrier with the data to be sent. It is a cost-effective communication technology as it can be installed using the present infrastructure, i.e., there is no need for any rewiring or any network modification costs and it is more commonly used in remote locations where cellular coverage is not readily available. Despite its cost-effectiveness, traditional PLCC networks generally have low bandwidths (in the range of 20 kbps) and are prone to noise and data distortion [87], which limits them to low-bandwidth applications, e.g., turning lights and appliances ON and OFF, etc. To overcome these limitations, a variety of PLCC variations such as Insteon (data rate and range of up to 13 kbps and 3000 m, respectively), IEEE 1901—HomePlug AV and HomePlug PV (data rate and range of up to 200 Mbps and 300 m), CE bus (data rate and range of up to 10 kbps and 3000 m) and LonWorks (data rate and range of up to 1.25 Mbps and 3000 m) have also been brought to the market in recent years [88,89].

### 3.3. Serial Communication and Modbus

Serial communication (the process of transmitting/receiving one bit at a time sequentially) via serial interfaces, such as RS232, RS422 and RS485, is the de-facto standard for wired inter-device communication today, which is attributed to its simplicity, high data rates (1–10 Mbps), range (up to 1200 m for RS422 and RS485, and 15 m for RS232), and reliability. RS232 has a single-ended mode of operation and allows for one driver and receiver on each line, whereas both RS422 and RS485 have differential modes of operation and allow for one driver/10 receivers and 32 drivers/32 receivers, respectively, making this medium well suited for nearly every present-day electronic device involving data communication [90].

Modbus, a serial communication protocol that supports RS232, RS422, RS485 and Ethernet, was first developed by Modicon (now Schneider Electric) in the mid-1970s for point-to-point communications between its programmable logic controllers (PLC). Decades later, it has now become one of the most frequently adopted methods for communication over serial lines between electronic devices, which is ascribed to its reliability, open-source availability and a simple design protocol based on Master/Slave communication where the Master is in full control on the bus and the Slave only responds when a request is sent by the Master. The standard Modbus design includes three related protocols, i.e., Modbus-RTU (Remote Terminal Unit), Modbus TCP (Transmission Control Protocol), and Modbus-ASCII (American Standard Code for Information Interchange), and in terms of typical applications, the three most common ones include: (1) controller/monitor to one smart device, (2) controller/monitor to multiple smart devices from the same vendor, and (3) remote monitoring of information from one smart device [91].

### 3.4. Zigbee

Zigbee (IEEE 802.15.4) is a global networking standard designed to use low-power digital radio signals for wireless personal area networks (WPAN). The technology operates in the 2.4 GHz and 900 MHz bands, supports star, tree and mesh topologies (enabling enhanced scalability, high reliability and a potentially broader coverage range), and is typically used to create communication networks that require a low data transfer rate (maximum of 250 kbps), and low power networking over a short distance (10–100 m) [92,93]. A Zigbee network consists of three types of nodes, i.e., Zigbee Coordinator (Full-Function Device (FFD)) responsible for initializing a session, selecting frequency, and managing security), Zigbee Router (FFD responsible for relaying messages to other nodes) and Zigbee End Device (Reduced-Function Device (RFD)) able to send and receive messages but perform no other special tasks. Despite limitations, such as low data rate and short range, Zigbee finds its applications in key areas within buildings such as security systems, building automation systems (BAS), and for smart meter and sensor readings, which is attributed to its high reliability, security, low power consumption and very low cost in comparison to other technologies. Moreover, Zigbee Alliance, an association of companies offering wireless communication solutions worldwide, have developed a wide range of standards, such as Zigbee Building Automation, Zigbee Remote Control, Zigbee Smart Energy, Zigbee Smart Energy Profile 2 and Zigbee Home Automation, to cater for different user requirements with regards to building applications [94,95].

### 3.5. Bluetooth and Bluetooth Low Energy (BLE)

Bluetooth (IEEE 802.15.1) is another wireless technology that was first developed by Ericsson in the mid-1990s and is used for communications over short distances (<10 m) at high data transfer rates (up to 2–24 Mbps). Bluetooth technology operates in the 2.4 GHz unlicensed ISM (Industrial, Scientific and Medical) frequency band, consists of 79 RF channels, 1 MHz channel bandwidth, a maximum of eight nodes for communications and consists of two main topologies, i.e., Piconet (formed by a WPAN and consisting of one mobile device assigned as Master and other mobile devices acting as Slaves), and Scatternet (consisting of two or more Piconets) [96,97]. Despite advantages such as high data rates and widespread availability, the technology currently has a number of drawbacks such as a short range of operation, limited number of nodes, relatively high-power consumptions (up to 50 mA during transmission [97]), and security and interference issues (as it has no strong security layer to prevent unauthorized interference, and operates on the same frequency range (2.4 GHz) as some other wireless technologies, i.e., Zigbee, Wifi, etc.). However, Bluetooth has been a widely adopted technology within buildings for several years, although its applications are limited to low-level data communications in mobiles and other portable devices, smart home appliances, computer peripherals and smart meters.

To overcome the power consumption limitations of the classic Bluetooth technology, a low-energy distinctive feature of the Bluetooth version v4.0 called Bluetooth Low Energy (BLE) was introduced by the Bluetooth SIG (Special Interest Group) [98]. BLE operates in the same frequency range, operates in the same Master–Slave model, and has the same number of nodes for communication as its classic counterpart. However, BLE has a larger coverage range (up to 50 m), and significantly lower energy consumptions (estimated to be up to 50% less) as it essentially operates in Sleep mode and only wakes up when a connection is initiated. These features make BLE well suited for applications such as building automation systems which only require periodic data transmissions with low-energy consumptions and fast data rates (see Table 3).

### 3.6. Wifi

WiFi (IEEE 802.11) is one of the most widely adopted IP-based wireless technologies in buildings worldwide, which is attributed to its key features such as high data rates, scalability, and existing global support, i.e., nearly all modern-day electronic devices (laptops, mobile phones, game consoles, etc.) contain WiFi communication capabilities. The technology is based on five main IEEE technology standards: 802.11a (54 Mbps), 802.11b (11 Mbps), 802.11g (54 Mbps), 802.11n (300 Mbps), 802.11ac (>1 Gbps), and can operate in three different unlicensed ISM frequency bands: 2.4 GHz, 3.5 GHz and 5 GHz. It is worth highlighting that channel assignment in WiFi varies in different countries around the world, e.g., the number of assigned channels in Europe is 13 whereas in the US it is 11, and therefore, only a limited number of devices can be connected in a WiFi network (due to the limited number of channels that can be used without an overlap) [93,99]. The maximum limit on the number of nodes in a WiFi network is 255 and the maximum coverage range for WiFi is typically up to 50–70 m, which, along with high power consumptions and interference challenges (as it operates on the same 2.4 GHz frequency band as other technologies such as Bluetooth and Zigbee), are some of the limitations of this technology. However, WiFi finds its applications in key areas such as building automation systems, meter readings, security systems, and communications between various electronic devices, such as laptops, televisions, mobile phones, etc. Moreover, a new WiFi standard called WiFi Direct has also been introduced in the last few years which enables devices to connect to each other without the need for a wireless access point (i.e., it essentially embeds a software access point into any device that supports this standard and, as a result, it bypasses the need to connect to a home, office or hotspot network) [100].

### 3.7. Other State-of-the-Art Communication Technologies

In addition to the aforementioned established communication technologies, other burgeoning technologies such as EnOcean [101], BACnet [102], 6LoWPAN [103], Z-Wave [104] and LoRaWAN [105] have also started to make their mark for building applications in recent years (see Table 3).

EnOcean is a wireless energy harvesting technology which combines micro energy converters with ultra-low power electronics to enable transmitters to harvest their energy from the environment, i.e., ambient light, temperature, motion, etc. This energy harvesting makes the technology well suited for wireless and battery-less sensors and switches used in building automation systems.

BACnet (Building Automation and Control Networking) is a standardized data communication protocol developed specifically for multiple devices to communicate across building automation systems by system users and building system manufacturers for applications such as HVAC, lighting control, fire access and security systems. The BACnet protocol services include Who-is, I-am, Who-has, I-have which are used for Object and Device discovery, and the standard defines a number of physical layers such as Ethernet, BACnet IP/IPv6, RS232/RS485 and Zigbee for different user requirements.

6LoWPAN (IPv6 over Low Power Wireless Personal Area Networks) is a wireless networking technology that optimizes IPv6 for use with low-power communication technologies such as IEEE 802.15.4 radios, hence enabling data packets to be carried efficiently within small link-layer frames at low bandwidths. In particular, 6LoWPAN takes advantage of the strong AES-128 link security within IEEE 802.15.4 and is considered well suited for applications such as building automation with sensors and actuators, street/residential lighting and control, and smart metering.

Z-Wave is another proprietary wireless technology designed specifically for the remote monitoring and control of residential and light commercial applications and has gained popularity in recent years due to the fact that the Z-Wave RF system operates in the sub-GHz (900 MHz) frequency range (unlike WiFi and other IEEE 802.11-based technologies), hence enabling a longer coverage range and lower power consumptions.

Finally, LoRaWAN, the latest of all available wireless technologies, is a low-power wide-area network (LPWAN) specification developed by LoRa Alliance for connecting wireless battery-operated devices in a regional, national or global network. Attributed to its long range (up to 10,000 m) and capability for a very large number of nodes (2000–3000) to be connected, LoRaWAN is now beginning to gain popularity for a wide range of smart building applications requiring secure bi-directional communication, localization services and mobility.

## 4. Battery and Energy Harvesting Technologies for Sensing and Monitoring in Buildings

### 4.1. Characteristics and Limitations of Battery Technologies

Batteries are an integral part of almost every technological aspect of our daily lives and continue to be one of the leading candidates to provide energy for sensing and monitoring devices used within modern-day buildings. This is attributed to features such as portability, high power densities (in most cases), and the sheer variety of technologies currently available on the market. Out of all the established technologies, Nickel Cadmium (NiCd), Nickel Metal-Hydride (NiMH), Nickel Iron (NiFe), Lead Acid, Lithium-ion (Li-ion), and Reusable Alkaline, have been the fastest growing, and most extensively studied and deployed technologies [106,107,108,109,110]. Table 4 provides a comparison of each of these battery technologies with data extracted or calculated from the latest published literature, and various manufacturer datasheets and user manuals available online.

It is evident from the data presented in Table 4 that each battery technology comes with its own set of distinct benefits, e.g., NiCd batteries have fast charge times, NiMH batteries have high power densities, NiFe batteries have a relatively low impact on the environment, and Li-ion batteries have the highest power densities of all the widely adopted technologies. However, batteries generally have a number of limitations which restrict them from meeting all the energy requirements of today’s sensing and monitoring devices deployed within buildings. These limitations include:

(i) Lifespan and Replacement—The lifetime of sensing and monitoring devices is always longer than the lifetime of the battery and, therefore, at some point, battery replacement is required (depending on the lifespan of the selected battery technology).
(ii) Self-discharging—Batteries self-discharge over time, which is caused by internal chemical reactions. Depletion due to self-discharging leads to reductions in the lifespan of the battery.
(iii) Size—Battery size usually exceeds the size of most sensing and monitoring devices.
(iv) Cost—Despite significant advancements in recent years, the cost of batteries continues to be higher than the cost of most sensing and monitoring devices summarized in Table 2 and Table 3. This cost increases further when more than one battery is required for a particular application (e.g., connecting multiple batteries in series or parallel for larger voltage requirements).
(v) Installation—Most batteries are able to operate in any position and provide good resistance to shocks and vibrations. However, in some cases, batteries have to be positioned on shock-absorbing dampers and in upright positions.
(vi) Disposal—Common battery technologies such as NiCd and Lead Acid contain environmentally hazardous materials and cannot be disposed with regular household items. Although NiMH and Lithium-based batteries are more environmentally friendly, it is still recommended to recycle all used batteries appropriately.
(vii) Maintenance—Apart from Li-ion and Alkaline, all other widely used battery technologies require regular maintenance every 30–180 days depending on usage.

To address the above restraints, the concept of ‘energy harvesting’, i.e., the process of converting ambient environmental energy into electrical energy to power small, low-power electronic devices, has become increasingly appealing in recent years. In the following subsections, we highlight some of the main sources of energy harvesting within buildings and discuss the technology’s potential viability for sensing and monitoring applications in buildings.

### 4.2. Potential of Building Energy Harvesting Technologies

In 2012, the World Economic Forum’s Global Agenda Council on Emerging Technologies highlighted ‘Wireless (Energy Harvested) Power’ as one of the top 10 technologies that would have the “greatest impact on the state of the world in 2012” [111]. The technology offers several unique features which conventional battery technologies cannot always provide, such as self-sustainable capability, prolonged lifetimes, no need for labor-intensive replacements, and easy installations [112]. However, at present, energy harvesting suffers from low, intermittent and unpredictable levels of available power, which limits its application to low-power electronic applications within buildings, e.g., low-power sensors, low-power rechargeable batteries, etc.

The natural sources of energy available for harvesting in buildings essentially consist of five main forms [113,114,115,116]:

(i) Photovoltaic (PV)—PV or solar cells convert ambient light energy (either natural sun light or artificial light) into electrical power and are the most mature and commonly used energy harvesting technology today. The amount of power harvested in PV energy harvesting is directly dependent on the incident angle of the light, intensity of the source, and the surface, size, sensitivity and material of the PV cell.
(ii) Thermal—Thermal (or Thermoelectric) energy harvesters convert heat into electrical power. These harvesters consist of arrays of thermocouples which sense a difference in temperature across their bimetal junctions (Seebeck effect) and produce a voltage in response to that.
(iii) Kinetic—Kinetic energy harvesters convert ambient mechanical energy (typically vibrations) into electrical power. Most kinetic harvesters, to date, convert vibrations into electrical energy using one of three main mechanisms, i.e., (1) electromagnetic (EM), (2) piezoelectric, and (3) electrostatic.
(iv) Radio Frequency (RF)—RF energy harvesters convert electromagnetic waves naturally available in the environment into electrical power. These RF waves are typically emitted from devices such as WiFi routers, radio and TV transmitters, mobile communications to and from base stations, etc.
(v) Airflow—Airflow energy harvesters use the ambient air generated by air conditioners, etc., inside buildings and convert it into electrical power. This is typically achieved via one of three methods, i.e., (1) wind turbine approach, (2) vibrating ribbon approach, and (3) cantilever approach.

**Figure 4:**
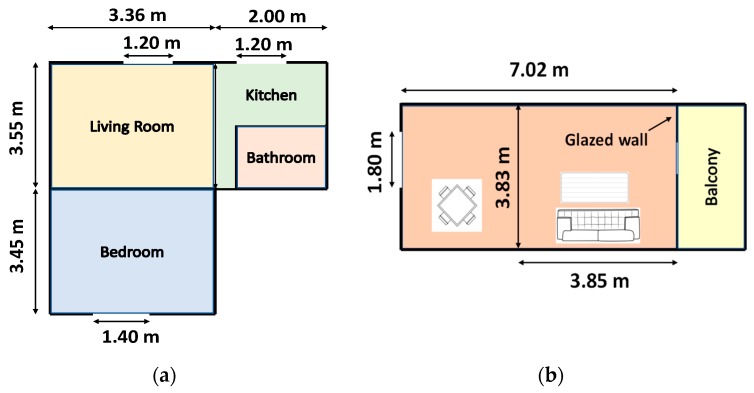
(**a**) Floor plan of a one-bedroom apartment in Southampton, UK. (**b**) Typical apartment living room configuration in San Sebastian, Spain [115].

To assess the performance of the aforementioned energy harvesting technologies, Matiko et al. [115] presented detailed surveys of different residential, commercial office and university buildings in three different European locations; (a) Southampton, UK (Figure 4a,b) San Sebastian, Spain (Figure 4b), and (c) Warsaw, Poland, and published measurements for light, temperature, vibrations, RF and airflow within these buildings. Table 5 provides a summary of the PV, Thermal, Kinetic, RF and Airflow measurements, i.e., estimated electrical dc power, and the calculated power densities of each energy source, for all the above-mentioned locations in [115]. It can be observed that Thermal and Airflow have the highest electrical dc powers (mW range) and power densities (mW/cm^3^ range) of all the energy harvesting technologies followed by PV and kinetic (µW range), and finally RF (nW range). However, technologies such as Thermal and Kinetic energy harvesting are heavily reliant on the working of household appliances such as radiators, boilers, washing machines, etc., which does not guarantee a long-term supply of available energy throughout the day. In this regard, PV energy harvesting is regarded as the most viable of all technologies at present due to the frequent availability of light (both natural and artificial) on a daily basis (up to 15–16 h a day), easy installations, no requirement for replacements and maintenance, and the overall maturity and variety of PV panel technologies in general, factors which make it the most attractive candidate of all for harvesting energy in buildings [117].

### 4.3. Commercial Building Energy Harvesting Solutions

From a commercial perspective, there are a number of energy harvesting solutions available off-the-shelf at present for potential use in buildings. EnOcean, its white label brand Dolphin, and the EnOcean wireless standard (discussed in Section 3), has enabled manufacturers to develop energy harvesting solutions for use in building management systems. Although EnOcean and NISSHA appear to be the two prominent manufacturers currently offering turnkey solutions; combining energy harvesting, sensors and data transmission in one pre-packaged unit, there are an increasing number of component manufacturers offering energy harvesting S °Cs and hardware. Table 6 highlights and compares some of these commercial energy harvesting solutions based on their respective energy conversion technologies, relevant characteristics and cost. The data has been extracted or calculated from various manufacturer datasheets and user manuals available online at the time of publication.

## 5. Discussion: Application, Challenges and Future Perspectives

### 5.1. Selection Parameters for Sensors and Communication Technologies in Buildings

It is evident from our detailed review in Section 2 and Section 3 that there is a plethora of sensing and communication technologies currently available on the market to cater for various requirements, and each one of them has its own respective sets of operating principles, characteristic features, and pros and cons. Whilst the arrangement, deployment costs, and other associated implementation challenges of sensor networks for different building applications may vary significantly based on variable factors such as location, size of building, number of devices and preferred technologies, they share an extensive set of common issues. As a result, their selection forms a design space which can consist of at least 13 different critical parameters which must be taken into account. The following key parameters can act as a checklist for building stakeholders, such as designers, managers, owners and occupants, considering the acquisition of various sensing and communication technologies for their respective applications.

(i) Heterogeneity—a single device/technology or a heterogeneous group with varying properties and hierarchies.
(ii) Cost—purchasing, installation and maintenance.
(iii) Availability—readily available for installation and replacement when required.
(iv) Deployment—once, incremental or random.
(v) Maintenance—monthly, quarterly, annually or maintenance-free.
(vi) Connectivity—continuous, occasional or sporadic.
(vii) Accuracy/Range—adequate accuracy and measurement/coverage range for desired application.
(viii) Security—vulnerability to any potential hacking/viruses/noise/interference.
(ix) Lifetime—a few hours, many months or several years.
(x) Power Consumption—low consumption to ensure long battery lifetimes, low energy bills etc.
(xi) Reliability—susceptibility to any potential disconnections/loss of power during data acquisition/storage/transmission.
(xii) Ruggedness—suitability for indoor/outdoor environments.
(xiii) Portability—flexibility to adjust/move network if and when required.

### 5.2. Optimal Placement of Sensors in Buildings

**Figure 5:**
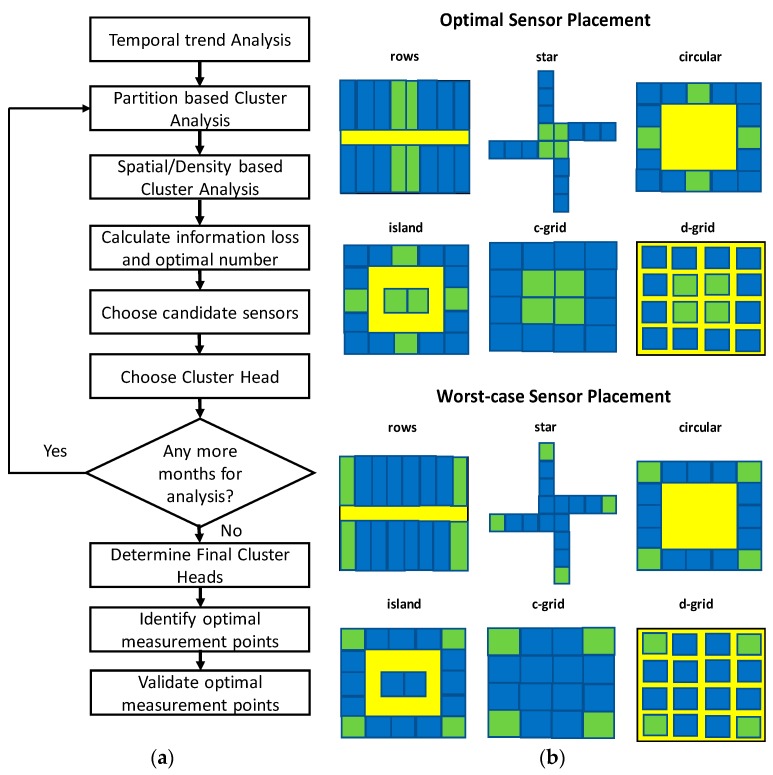
(**a**) Systematic approach presented by Yoganathan et al. [119] for optimal sensor placement within office buildings using clustering algorithms, data loss and the Pareto principle. (**b**) Best- and worst-case placements for four sensors (highlighted in green) in a 16-room building considering six different typically used building topologies [123].

In addition to the most conducive selection of sensor devices, their optimal placement within buildings is equally important. Generally, a larger number of sensors and monitoring technologies are preferred as this provides a more accurate representation of the various physical parameters inside the building. However, this undoubtedly has a direct impact on critical factors such as cost, timelines and complexity (due to additional installations), deployment, commissioning, maintenance and data processing required as part of this process. To address this challenge of reducing the number of deployed sensors and making best use of the space available, several optimization strategies and mathematical models for sensor placement have been presented in recent years. Eliades et al. [118] proposed a methodology for the placement of indoor air quality sensors for air contamination prevention, firstly, by formulating a multi-objective optimization problem for minimizing sensor cost, average and worst-case impact damage corresponding to a set of contamination event scenarios, and secondly, by constructing a set of probability-distribution-representative contamination scenarios through grid and random sampling to compute the overall impact of each scenario. The application of the proposed methodology on two different buildings, i.e., a five-room and a 14-room building, and a comparison of the average and worst-case impact showed that as the number of installed sensors is increased, the change in the impact risk objectives is reduced and may not be significant. Yoganathan et al. demonstrated in [119] that by adopting cluster algorithms, data loss approach, and the Pareto principle (also referred to as the 80/20 rule, i.e., for many events 80% of the effects come from 20% of the causes), the optimal number and location of light, humidity and temperature sensors in their case study building could be determined. The systematic approach adopted by the authors (shown in Figure 5a) revealed that the number of deployed sensors can be reduced to 20%, (i.e., from 31 down to 6) with minimum loss of information. Other studies using techniques such as a maximum-likelihood estimator [120], Fisher information matrix [121] and Information Entropy Norm [122] have also proposed sensor placement strategies. However, each of these mentioned techniques determine the placement of sensors in a heuristic manner which does not necessarily provide guaranteed optimum generalizable solutions.

In ref. [123], T. Seabrook proposed a generalized optimal sensor placement strategy for minimum effective sensors by considering four sensors to be placed in a 16-room building with six different typically used building topologies given below:

(i) Rows—two rows of rooms in a corridor.
(ii) Star—rooms meet at a central point.
(iii) Circular—all rooms have two neighbours and surround a central space.
(iv) Island—Similar to circle but with rooms in the central space.
(v) Compact-Grid—All rooms are connected with no corridors.
(vi) Dispersed-Grid—All rooms are disconnected by a corridor.

The best- and worst-case placement results for this study have been depicted in Figure 5b, where it is shown that it is more favorable to place sensors in central positions within the building as opposed to dispersed positions. The Row and Star topologies highlight that it is favorable to place sensors in rooms with the highest number of connected neighbours to minimize the number of neighbours that do not have sensors, reducing the complexity of correction-less predictions, and to offer an overall systematic placement to maximize the coverage. The Circular, Compact-Grid (c-grid) and Dispersed-Grid (d-grid) topologies show that placement is favorable where higher connections to corridors exist as opposed to outside walls, hence supporting the placement of sensors in more typical rooms (as there are, on average, more inner walls per room than outer). Finally, the Island topology highlights that (at least one) sensor should be placed in each isolated group if possible [123].

### 5.3. Routing Protocols for Energy Efficiency Enhancement

Power consumption and energy conservation within sensor networks has been a critical issue for a number of years as sensor nodes are typically powered by various battery technologies which, as discussed in Section 4, can have limited lifespans and other constraints such as cost, installation complexities, maintenance and size. Therefore, energy-efficient design for sensor networks has been a focal point of research and development and has resulted in several energy-efficient routing (EER) protocol approaches being presented [124,125,126,127]. These protocols can largely be categorized into three main classifications, i.e., (i) Hierarchical (or Clustering-based), (ii) Data Centric, and (iii) Location-based (Geographical), although other classifications such as (iv) Data Relaying, (v) Mobility-based, and (vi) Heterogeneous protocols have also been proposed in the literature.

(i) Hierarchical protocols—in these protocols the sensor network is broken down into clusters with multiple gateways where each sensor node within a cluster communicates with other nodes in a multi-hop manner, leading to more efficient energy consumption. Some of the prominent Hierarchical protocols include LEACH (low-energy adaptive clustering hierarchy) [128], PEGASIS (power efficient gathering in sensor information systems) [129], TEEN (threshold sensitive energy efficient network) [130] and APTEEN (adaptive threshold sensitive energy efficient network) [131].
(ii) Data-Centric protocols—in these protocols, a set of sensor nodes can be selected based on a query-based model and data can also be acquired whilst being transmitted to the base station (i.e., the sink node where data is collected and processed). Some of the prominent Data-Centric protocols include SPIN (sensor protocols for information via negotiation) [132] and Directed Diffusion [133].
(iii) Location-based (Geographical) protocols—in these protocols, the location information of sensor nodes is required in order to calculate the distance between two particular nodes and draw the most energy-efficient path between them. Some of the prominent Location-based protocols include GAF (geographic adaptive fidelity) [134], GEAR (geographic and energy-aware routing) [135] and LEAR (location-based energy-aware routing) [136].

In Table 7, we present a comparison of some of the aforementioned routing protocols in terms of key performance characteristics such as scalability, power management, mobility, resource awareness, data aggregation capabilities and network lifetime.

The performance of various routing protocols is directly dependent on the design of the sensor network, and in this regard, there are several challenges at present, such as scalability, mobility, energy consumption, data aggregation, network lifetime, delay times and quality-of-service (QOS), which must be overcome for optimum results. To address these challenges, we can expect future research to focus on vital areas such as application-specific routing protocol development, more rigorous mathematical modelling and simulations, and hardware experimentation on real test beds to address any shortcomings within mathematical models and simulations [126]. Other key aspects for future research include making sensor networks and routing protocols increasingly self-sufficient (i.e., limiting the need for human intervention for network configuration, calibration, maintenance and repair), further advancements in memory and storage space for data collection and processing, and enhancing fault tolerance within sensor networks (i.e., ensuring that networks continue to function even if certain nodes within the network fail during operation).

### 5.4. Data Analysis and Network Security

Within the operational phase, buildings are now producing more data than ever before, and this data can be leveraged to reduce the energy consumption and operational costs of the building [137]. Datasets can provide useful information relating to energy, demand, occupancy, weather and pricing, that can be used to aid the development of demand-side response techniques, appropriately schedule building loads, maximize profit from peer-to-peer energy trading, or optimize the performance of energy harvesting–battery systems.

Managing and creating value from large volumes of data can be a cumbersome task which involves communicating collected data over a network, storing data in database systems, cleaning and filtering data to identify missing, erroneous or duplicate values and applying machine learning techniques to make predictions and classifications [138]. The granularity of datasets required to capture transient behavior of certain parameters can be very high, and therefore, data processing can be an intensive and energy-consuming process. This data can be processed locally where it is collected so that no raw data is communicated to the remote server—increasing the overall energy performance by reducing communication needs, and can also be processed on the ‘edge’ of a network, where network devices are utilized to store and process data, or on the cloud, which provides highly scalable computing resources as a pay per use service [139]. In the future, we can expect further growth in these approaches as the sheer volume of data acquired within buildings continues to increase.

Some of the prominent challenges associated with unlocking value from building datasets which need to be overcome in the future include the lack of a common data structure—such as database schema and naming methodologies—which limits the interoperability of information between buildings. This has been recognized by experts and is currently the driver behind the ASHRAE 223P specification for standardized building schema [140,141]. The security of the sensor networks is also a concern as sensors can become points of intrusions, malicious attacks and other cyber threats [142]. This problem is particularly acute when public networks are used to transmit data.

### 5.5. The Future of Building IOT (Internet of Things) Technologies

The sensor and communication technologies discussed in previous sections of this review reside at the forefront of ‘Internet of Things (IoT)’, comprised of a network of interconnected devices including sensing and communication technologies, and associated software and electronic hardware instrumentation, which enable these devices to rapidly acquire, process, and transmit and receive data over fast networks. IoT sensors, also referred to as ‘smart sensors’, are able to not only detect and output physical events and changes in the environment but also generate much larger amounts of data with increased granularity and communicate the acquired information efficiently throughout a connected domain.

Recent reports have forecasted that the number of IoT-connected devices, sensors and actuators are expected to exceed 46 billion by 2021 [143] and the IOT market has a total potential economic impact of $3.9 trillion to $11.1 trillion a year by 2025 [144]. Despite these exciting propositions, its adoption and implementation in the building sector has been slower than expected so far. As discussed in the previous sub-section, the security of sensor networks is a significant challenge at present as the network becomes more prone to hacking or loss of data, and restricts data privacy and sharing, when billions of sensor and communication devices are connected together over the internet. At the same time the storage, tracking and analysis of the sheer amount of data produced by billions of devices becomes increasingly challenging. Key factors such as device scalability, cost and power consumption, installation complexities, selection and placement of sensing and communication technologies for optimum coverage and performance, and some manufacturers keeping their devices proprietary to other hardware for market dominance, also continue to hinder the wide-scale adoption of the technology at present. This highlights the need for the integration of all-intelligent systems and digital networks within buildings which can enable wide-range networking, digitization, and the capability to collectively acquire and analyze data to make real-time building-wide decisions. In the future, we can expect an increase in cutting-edge intelligent sensor technologies consisting of low-cost, low-power processors and inbuilt energy harvesting capabilities. Furthermore, enhanced embedded support for various communication protocols, and further development of intuitive machine learning algorithms and user-friendly software applications will also play an essential role in addressing the aforementioned challenges in the future.

### 5.6. The Future of Building Energy Harvesting

Our discussions in Section 4 indicate that energy harvesting is expected to play an increasingly important role in powering various sensing and monitoring devices within the buildings of the future. Although these technologies have significantly lower power densities at present (Table 5), especially when compared to those for batteries (Table 4), in most cases, these power densities (in the mW range), matched with efficient energy accumulation mechanisms, can be sufficient for powering various sensor devices, recharging batteries, and powering other electronic appliances within buildings. Despite these limitations at present, the technology provides some vital benefits such as self-sustainability, prolonged lifetimes, ease of installation, and a multitude of available ambient sources of energy. Distinctive approaches to energy harvesting, such as hybrid energy harvesters (that utilize multiple sources of power) [145], harvesting energy from water and soil pH difference [146,147], tree trunks and leaves [148,149], and using graphene-based materials and structures [150], have also been explored in recent years, indicating the future potential for less conventional energy harvesting sources. Demonstration projects for building-integrated renewable technologies (e.g., SPECIFIC’s ‘Active Classroom’ and ‘Active Office’ [12] (Figure 1a) which utilize a wide range of sensor, communication, battery and renewable energy technologies will also continue to play a primary role in proving reliability and capability in use, validating theories and models, and ensuring that the building sensors and controls are usable and not overly complicated for all technical and non-technical stakeholders involved.

## 6. Summary and Conclusions

In this paper, we have presented an in-depth review of the state-of-the-art of different sensing and environmental monitoring technologies commercially available and widely used today for building applications. By surveying published literature, manufacturer datasheets and user manuals available at the time of publication, we (1) identify the most essential and prominent sensor technologies as: temperature, carbon dioxide, humidity, occupancy, light and airflow measurement technologies, and communication technologies and protocols (both wired and wireless) as: Ethernet, PLCC, Serial Communications, Modbus, BACnet, Zigbee, Bluetooth, BLE, WiFi, EnOcean, 6LoWPAN, Z-Wave and LoRaWAN, and (2) provide a detailed insight into their operating principles and key characteristic features, such as accuracies, measurement ranges, response times, portability, power consumptions, market adoption and costs.

In addition to the detailed survey of various sensor and communication technologies, we also (1) identify the most prominent battery technologies used for sensing and monitoring purposes as: Nickel Cadmium, Nickel Metal Hydride, Nickel Iron, Lead acid, Lithium-ion and Reusable Alkaline, (2) review their key characteristics, such as cycle life, lifespans, self-discharge rates, charge times, environmental impact, maintenance requirements, costs, and power densities, and (3) highlight various time-related limitations of battery technologies, such as self-discharging, size, cost, installation, disposal, maintenance and the eventual need for replacement. In the last decade, energy harvesting from ambient sources within buildings has also emerged as a leading candidate to address the limitations of battery technologies and use the harvested electrical energy to power small low-power electronic devices. We (1) identify the five main sources for energy harvesting in buildings as: Photovoltaic, Thermal, Kinetic, Radio Frequency and Airflow, (2) highlight the potential of each of these sources by discussing the findings of a real-world building case-study conducted in three different European countries, i.e., UK, Spain and Poland, and (3) review various commercial off-the-shelf energy harvesting solutions available for building applications. The literature suggests that power densities generated from building energy harvesting are significantly lower than those available from various battery technologies. However, these power densities (in the mW range) can be sufficient to power low-power sensors and monitoring devices, and recharge batteries used in buildings.

In the final section of this review, we discuss different aspects regarding the application of sensing and monitoring technologies within buildings, and various associated challenges and future perspectives. The selection of the most suitable technologies can be a challenging task as there are a number of parameters that building designers, owners, managers, and occupants must take into consideration. We highlight 13 key parameters that must be taken into account, and review proposed strategies and topologies for the optimal placement of sensor devices within buildings. Moreover, we discuss key topics such as routing protocols for energy efficiency enhancement, data analysis and network security and the future of building IOT and energy harvesting technologies. The unique features that energy harvesting provides such as self-sustainable capability, prolonged lifetimes, no need for labor-intensive replacements, easy installation, and the variety of available natural sources of energy within buildings, makes energy harvesting one of the most attractive technologies for buildings of the future.

## Figures and Tables

**Table 1:** Summary of the key characteristics of temperature, carbon emission, and humidity sensors used in buildings. Data has been deduced or calculated from published literature, and various manufacturer datasheets and user manuals available online at the time of publication.

Parameter.	Sensor	Measurement Range	Accuracy	Response Time	Power Consumption	Applications/Technology	Cost
Temperature	Thermocouples	−100–500 °C	±1–4 °C	5–80 s	Low–High (0.5 µA–30 mA)	BMS, HVAC/Wired, Portable	$6–50
	RTDs	−50–250 °C	±0.2–1 °C	1–8 min	High during measurement (1.5–100 mA)	BMS, HVAC and Visualisation/Wired, Wireless	$30–100
	Thermistors	−50–130 °C	±0.05–0.5 °C	0.2–10 s	High during measurement (1–80 mA)	BMS, HVAC and Visualization/Wired, Wireless	$20–70
	IC sensors	−40–150 °C	±0.5–1 °C	0.5–100 s	Low (0.5–100 µA)	BMS, HVAC and Visualisation/Wired, Wireless	$1–15
Carbon emissions	NDIR (CO_2_)	0–10,000 ppm	±30–200 ppm	30–100 s	Low–High (20–200 mA)	Airflow Control, Monitoring/Wired, Wireless	$100–600
	MOSFET (CO and VOC)	400–20,000 ppm	±30–100 ppm	50–60 s	High (typ. >50 mA)	Airflow Control, Monitoring/Wired, Wireless	$25–250
	Electrochem. (CO and VOC)	0–1000 ppm	±0–30 ppm	10–60 s	Low (30 µA–10 mA)	Airflow Control, Monitoring/Wired, Wireless	$100–650
Humidity	Capacitive sensors	0%–100% RH	±0%–5%	15–90 s	Low (2 µA–4 mA)	BMS, HVAC and Visualisation/Wired, Wireless	$40–200
	Resistive sensors	5%–90% RH	±1%–10%	10–60 s	Low (0.5–5 mA)	BMS, HVAC and Visualisation/Wired, Wireless	$25–170

**Table 2:** Comparison of the key characteristics of airflow, light, and motion detection sensors used in buildings. Data has been extracted or calculated from published literature, and various manufacturer datasheets and user manuals are available online at the time of publication.

Parameter	Sensor	Measurement Range/Accuracy	Response Time	Power Consumption	Applications/Technology	Suitability for both Indoor and Outdoor Environments	Cost
Occupancy	PIR Ultrasonic	3–10 m distance >70 m	0.5 s–10 min 1.3 ms–30 min	Low–High (5–15 mA) Low–High (5–30 mA)	BMS, HVAC and Visualisation/Wired, Wireless BMS, HVAC and Visualisation/Wired, Wireless	No Yes	$20–65 $130–500
Light	Photores. Photodiode	±5%–10% of reading ±5%–10% of reading	5–20 s 1–10 s	Low–High (10–60 mA) Low (100 µA–5 mA)	BMS, HVAC and Visualisation/Wired, Wireless BMS, HVAC and Visualisation/Wired, Wireless	Yes Yes	$50–200 $15–65
Airflow	Hotwire Anemom. Vane Anemom.	0.1–45 m/s air velocity 0.25–50 m/s air velocity	0.1–5 s 0.5–5 s	Low–High (10–40 mA) Low–High (2–50 mA)	HVAC, Airflow control and monitoring/Wired, Wireless HVAC, Airflow control and monitoring/Wired, Wireless	No Yes	$25–200 $25–300

**Table 3:** Key characteristics of prominent wired and wireless technologies for building applications.

Technology (Standard)	Coverage Range	Theoretical Data Rate	Maximum Number of Nodes	Power Consumption	Market Adoption
Ethernet (IEEE 802.3)	100 m	10 Mbps–100 Gbps	1 per wire (254 on a subnet)	Low	High
PLCC (Insteon, IEEE 1901, CE bus, LonWorks)	300–3000 m	13 kbps–200 Mbps	500–1000	Low–High	Low
Serial Comm. and Modbus (RS232, RS422, RS485)	15–1200 m	1–10 Mbps	32 typical. Up to 256 with some ICs	Low	High
Zigbee (IEEE 802.15.4)	10–100 m	250 kbps	255	Low	High
Bluetooth (IEEE 802.15.1) BLE	10 m 50 m	2–24 Mbps 2 Mbps	8 8	High Low	High Low
WiFi (IEEE 802.11 a,b,g,n,ac)	50–70 m	11–1300 Mbps	255	High	High
EnOcean (EnOcean standard)	20–200 m	125 kbps	2^32^	Low	Low
BACnet (ANSI/ASHRAE 135)	1200 m	9.6–115.2 kbps	32 typical. Up to 128 Master Nodes on same Segment	Low	Low
6LoWPAN (IEEE 802.15.4)	10–100 m	250 kbps	2^64^	Low	Low
Z-Wave (Z-Wave Alliance)	30–300 m	100 kbps	2^32^	Low	Low
LoRaWAN (LoRa Alliance)	10,000 m	0.3–50 kbps	2000–3000	Low	Low

**Table 4:** Comparison of the key characteristics of various battery technologies used for sensing and monitoring in buildings. Data has been extracted or calculated from published literature, and various manufacturer datasheets and user manuals are available online at the time of publication.

Parameter	NiCd	NiMH	NiFe	Lead Acid	Li-ion	Reusable Alkaline
Cycle Life (cycles)	1000–5000	300–2000	1000–8000	100–500	300–5000	10–100
Lifespan (years)	5–15	2–8	10–30	1.5–10	2–10	1–5
Self-discharge Rate (%/month)	15–20 (typ. decrease of 10% in first 24 h, then 10% for 30 days)	25–35	20–30	3–6	10%–15% (typ. 3% of energy consumed by internal circuit protection)	0.2–1
Fast Charge Time (hours)	1–2	2–4	4–6	8–14	2–4	1–3
Power Density (µW/cm^3^)	40,000–100,000	8000–500,000	10,000–30,000	10,000–350,000	60,000–800,000	10,000–100,000
Market Adoption	High	High	Medium	High	High	High
Environmental Impact	High	Medium	Low	High	Medium	High
Maintenance Requirement (days)	30–60	60–90	70–100	90–180	Not required	Not required
Battery Cost ($/kWh)	$250–550	$250–500	$180–250	$100–200	$200–400	$70–200

**Table 5:** Summary of recorded measurements and the overall power performance of five main energy harvesting technologies available in buildings (Matiko et al. [115]).

Energy Harvesting Source	Typical Range of Ambient Energy Levels	Estimated Electrical DC Power	Calculated Power Density
Photovoltaic	Light intensity: 100–3700 1x	25–1149 µW	9–399 µW/cm^3^
Thermal	Thermal gradient: 10–40 °C	1–10 mW	0.7–7.1 mW/cm^3^
Kinetic	Acceleration: 0.0245–2.82 m/s^2^ Frequency: 43.1–162.3 Hz	0.008–68.97 µW	0.05–459.8 µW/cm^3^
RF	EM wave strength: −74 to −29 dBm	0.028–944 nW	0.00169–57.37 nW/cm^3^
Airflow	Airflow speed: 1–10 m/s	0.9–324 mW	0.017–6.0 mW/cm^3^

**Table 6:** Comparison of various commercial energy harvesting solutions available for potential use in buildings.

Product ID	Harvesting Technology	Product Type	Manufacturer	Cost
‘Aerial Switch’	Photovoltaic	Turnkey product—HMI switch	NISSHA	POA
S3001-D330	Photovoltaic	Turnkey product—Temperature sensor	EnOcean	$27
S3001-D320	Photovoltaic	Turnkey product—Magnetic contact sensor	EnOcean	$30
EPACA	Photovoltaic	Turnkey product	EnOcean	n/a
AEM40940	RF	Power Management IC	E.PEAS	$4
STM300	n/a	Low-power transmitter module	EnOcean	$26
PCT100	RF via RFID (less than 10 m)	Light and temperature transmitter module	Powercast	$83
PMG17-100	Vibration-EM coil	Energy source	Perpetuum	POA
APA 400M	Vibration-Piezo	Energy source	Cedrat Technologies	POA
S233-H5FR	Vibration-Piezo	Energy source	Mide	$140
HZ-14	Thermoelectric (Seebeck)	Energy source	Hi-Z	$11
CZ1	Thermoelectric (Seebeck)	Energy source	Tellurex	$12
PL-ENO-SET1	Mechanical	Turnkey product—HMI switch	EnOcean	$100
EH300	Any intermittent electric source (0–500V AC/DC)	Energy source	Advanced Linear Devices	$80

**Table 7:** Comparison of some key characteristics of various routing protocols for sensor networks.

Protocol	Type	Scalability	Power Management	Mobility	Resource Awareness	Lifetime	Data Aggregation
LEACH	Hierarchical	High	Very Good	Sink node (base station) is fixed	Yes	Very Good	Yes
PEGASIS	Hierarchical	High	Very Good	Sink node (base station) is fixed	Yes	Very Good	Yes
TEEN	Hierarchical	High	Very Good	Sink node (base station) is fixed	Yes	Very Good	Yes
APTEEN	Hierarchical	High	Very Good	Sink node (base station) is fixed	Yes	Very Good	Yes
SPIN	Data-Centric	Low	Limited	Supported	Yes	Good	Yes
Direct Diffusion	Data-Centric	Low	Limited	Limited	Yes	Good	Yes
GAF	Location-based	Low	Limited	Limited	Yes	Good	No
GEAR	Location-based	Low	Limited	Limited	Yes	Good	No

## References

[1] BEIS (2016). Energy Consumption in the UK.

[2] DTI (2006). The Energy Challenge Review.

[3] Conti J., Holtberg P., Diefenderfer J., LaRose A., Turnure J.T., Westfall L. (2016). International Energy Outlook 2016 with Projections to 2040.

[4] Ahmad M.W., Mourshed M., Mundow D., Sisinni M., Rezgui Y. (2016). Building energy metering and environmental monitoring—A state-of-the-art review and directions for future research. Energy Build..

[5] Dean B., Dulac J., Petrichenko K., Graham P. (2016). Towards Zero-Emission Efficient and Resilient Buildings.

[6] Grözinger J., Boermans T., Wehringer A.J.F., Seehusen J. (2014). Overview of Member States Information on NZEBs: Background Paper—Final Report.

[7] Department of Energy and Climate Change (DECC). https://www.gov.uk/2050-pathways-analysis.

[8] The World Bank (2014). World Development Indicators 1960–2013.

[9] GAIA—“Green Awareness in Action”. gaia-project.eu.

[10] OrbEEt. https://orbeet.eu/.

[11] TRIBE: “Training Behaviours towards Energy Efficiency: Play It”. https://tribe-h2020.eu/.

[12] SPECIFIC—“Buildings as Power Stations”. https://specific.eu.com/.

[13] Chung M.H., Rhee E.K. (2014). Potential opportunities for energy conservation in existing buildings on university campus: A field survey in Korea. Energy Build..

[14] Council G.B. (2011). Carbon Reductions in Existing Non-Domestic Buildings.

[15] Menezes A.C., Cripps A., Bouchlaghem D., Buswell R. (2011). Analysis of electricity consumption for lighting and small power in office buildings. CIBSE Technical Symposium.

[16] Standeven M., Cohen R., Bordass B., Leaman A. (1998). PROBE 14: Elizabeth fry building. Build. Serv. J..

[17] Rajaram S. (2016). Global Markets and Technologies for Sensors.

[18] Johnson R.C. (2014). Roadmap to Trillion Sensors Forks—EE Times Dec. 2015.

[19] Mohassel R.R., Fung A., Mohammadi F., Raahemifar K. (2014). A survey on advanced metering infrastructure. Int. J. Electr. Power Energy Syst..

[20] Behrooz F., Mariun N., Marhaban M., Mohd Radzi M., Ramli A. (2018). Review of control techniques for HVAC systems—Nonlinearity approaches based on Fuzzy cognitive maps. Energies.

[21] Darby S. (2008). Energy feedback in buildings: Improving the infrastructure for demand reduction. Build. Res. Inf..

[22] Bichiou Y., Krarti M. (2011). Optimization of envelope and HVAC systems selection for residential buildings. Energy Build..

[23] Childs P.R.N., Greenwood J.R., Long C.A. (2000). Review of temperature measurement. Rev. Sci. Instrum..

[24] Cheng C.C., Lee D. (2016). Enabling Smart Air Conditioning by Sensor Development: A Review. Sensors.

[25] Doukas H., Patlitzianas K.D., Iatropoulos K., Psarras J. (2007). Intelligent building energy management system using rule sets. Build. Environ..

[26] Kolokotsa D., Pouliezos A., Stavrakakis G., Lazos C. (2009). Predictive control techniques for energy and indoor environmental quality management in buildings. Build. Environ..

[27] Kumar P., Martani C., Morawska L., Norford L., Choudhary R., Bell M., Leach M. (2016). Indoor air quality and energy management through real-time sensing in commercial buildings. Energy Build..

[28] OMEGA Temperature Sensors Website. https://www.omega.co.uk/subsection/temperature-sensors-instruments.html.

[29] Texas Instruments Website. https://www.e2e.ti.com.

[30] National Instruments Website. https://www.ni.com.

[31] Leephakpreeda T., Thitipatanapong R., Grittiyachot T., Yungchareon V. (2001). Occupancy-based control of indoor air ventilation: A theoretical and experimental study. Sci. Asia.

[32] Prill R. (2000). Why Measure Carbon Dioxide Inside Buildings.

[33] Mysen M., Rydock J.P., Tjelflaat P.O. (2003). Demand controlled ventilation for office cubicles—Can it be profitable?. Energy Build..

[34] Dougan D.S., Damiano L. (2004). CO_2_-based demand control ventilation: Do risks outweigh potential rewards?. ASHRAE J..

[35] Mumma S.A. (2004). Transient occupancy ventilation by monitoring CO_2_. ASHRAE IAQ Appl..

[36] Nassif N. (2012). A robust CO_2_-based demand-controlled ventilation control strategy for multi-zone HVAC systems. Energy Build..

[37] Yasuda T., Yonemura S., Tani A. (2012). Comparison of the characteristics of small commercial NDIR CO_2_ sensor models and development of a portable CO_2_ measurement device. Sensors.

[38] Senseair Website. https://www.senseair.com.

[39] Vaisala Website. https://www.vaisala.com.

[40] Amphenol Website. https://www.amphenol-sensors.com.

[41] Trotec Website. https//www.trotec.com.

[42] Lin C., Xian X., Qin X., Wang D., Tsow F., Forzani E., Tao N. (2018). High Performance Colorimetric Carbon Monoxide Sensor for Continuous Personal Exposure Monitoring. ACS Sens..

[43] Spinelle L., Gerboles M., Kok G., Persijn S., Sauerwald T. (2017). Review of portable and low-cost sensors for the ambient air monitoring of benzene and other volatile organic compounds. Sensors.

[44] Patil S.J., Patil A.V., Dighavkar C.G., Thakare K.S., Borase R.Y., Nandre S.J., Deshpande N.G., Ahire R.R. (2015). Semiconductor metal oxide compounds based gas sensors: A literature review. Front. Mater. Sci..

[45] Szulczyński B., Gębicki J. (2017). Currently commercially available chemical sensors employed for detection of volatile organic compounds in outdoor and indoor air. Environments.

[46] Mullassery D.J. (2015). Sensors and Analytics for Smart Buildings.

[47] Fraden J. (2004). Handbook of Modern Sensors: Physics, Designs, and Applications.

[48] Farahani H., Wagiran R., Hamidon M.N. (2014). Humidity sensors principle, mechanism, and fabrication technologies: A comprehensive review. Sensors.

[49] Roveti D.K. Choosing a Humidity Sensor: A Review of Three Technologies. https://www.fierceelectronics.com/components/choosing-a-humidity-sensor-a-review-three-technologies.

[50] OMEGA Humidity Sensors Website. https://www.omega.com/en-us/sensors-and-sensing-equipment/c/humidity.

[51] TE Connectivity Website. https://www.te.com.

[52] Honeywell Sensors Website. https://sensing.honeywell.com.

[53] Garg V., Bansal N.K. (2000). Smart occupancy sensors to reduce energy consumption. Energy Build..

[54] Yavari E., Song C., Lubecke V., Boric-Lubecke O. (2014). Is there anybody in there? Intelligent radar occupancy sensors. IEEE Microw. Mag..

[55] Oldewurtel F., Sturzenegger D., Morari M. (2013). Importance of occupancy information for building climate control. Appl. Energy.

[56] Yun J., Song M.H. (2014). Detecting direction of movement using pyroelectric infrared sensors. IEEE Sens. J..

[57] Labeodan T., Zeiler W., Boxem G., Zhao Y. (2015). Occupancy measurement in commercial office buildings for demand-driven control applications—A survey and detection system evaluation. Energy Build..

[58] Schneider Electric—Sensors Website. https://www.schneider-electric.com.

[59] Hubbell Sensors Website. https://www.hubbell.com.

[60] Ul Haq M.A., Hassan M.Y., Abdullah H., Rahman H.A., Abdullah M.P., Hussin F., Said D.M. (2014). A review on lighting control technologies in commercial buildings, their performance and affecting factors. Renew. Sustain. Energy Rev..

[61] Guo X., Tiller D.K., Henze G.P., Waters C.E. (2010). The performance of occupancy-based lighting control systems: A review. Lighting Res. Technol..

[62] Candanedo L.M., Feldheim V. (2016). Accurate occupancy detection of an office room from light, temperature, humidity and CO_2_ measurements using statistical learning models. Energy Build..

[63] Benezeth Y., Laurent H., Emile B., Rosenberger C. (2011). Towards a sensor for detecting human presence and characterizing activity. Energy Build..

[64] Huang Q., Ge Z., Lu C. (2016). Occupancy estimation in smart buildings using audio-processing techniques. arXiv.

[65] Pathak P.H., Feng X., Hu P., Mohapatra P. (2015). Visible light communication, networking, and sensing: A survey, potential and challenges. IEEE Commun. Surv. Tutor..

[66] Vishay Sensors Website. https://www.vishay.com.

[67] Panasonic Sensors Website. https://www.panasonic.com.

[68] Philips Sensors Website. https://www.philips.com.

[69] Mayer H., Höppe P. (1987). Thermal comfort of man in different urban environments. Theor. Appl. Climatol..

[70] De Dear R.J., Brager G.S. (2002). Thermal comfort in naturally ventilated buildings: Revisions to ASHRAE Standard 55. Energy Build..

[71] Saddoughi S.G., Veeravalli S.V. (1996). Hot-wire anemometry behaviour at very high frequencies. Meas. Sci. Technol..

[72] Adamec R.J., Thiel D.V., Tanner P. MEMS wind direction detection: From design to operation. Proceedings of the IEEE Sensors 2003.

[73] Caracoglia L., Jones N.P. (2009). Analysis of full-scale wind and pressure measurements on a low-rise building. J. Wind Eng. Ind. Aerodyn..

[74] Siemens Sensors Website. https://www.siemens.com.

[75] Testo Website. https://www.testo.com.

[76] Seton Website. https://www.seton.com.

[77] Labeodan T., De Bakker C., Rosemann A., Zeiler W. (2016). On the application of wireless sensors and actuators network in existing buildings for occupancy detection and occupancy-driven lighting control. Energy Build..

[78] Rodrigues F., Cardeira C., Calado J.M.F. (2010). The Impact of Wireless Sensors in Buildings Automation.

[79] De Farias C., Soares H., Pirmez L., Delicato F., Santos I., Carmo L.F., de Souza J., Zomaya A., Dohler M. (2014). A control and decision system for smart buildings using wireless sensor and actuator networks. Trans. Emerg. Telecommun. Technol..

[80] Kazmi A.H., O’grady M.J., Delaney D.T., Ruzzelli A.G., O’hare G.M. (2014). A review of wireless-sensor-network-enabled building energy management systems. ACM Trans. Sens. Netw..

[81] Grimard T., Kieran L. Wireless Options Becoming More Prevalent with BAS. https://www.facilitiesnet.com/buildingautomation/article/Wireless-Options-Becoming-More-Prevalent-with-BAS--16393.

[82] Jansen D., Buttner H. (2004). Real-time Ethernet: The EtherCAT solution. Comput. Control. Eng..

[83] Schemm E. (2004). SERCOS to link with ethernet for its third generation. Comput. Control. Eng..

[84] Kjellsson J., Vallestad A.E., Steigmann R., Dzung D. (2009). Integration of a wireless I/O interface for PROFIBUS and PROFINET for factory automation. IEEE Trans. Ind. Electron..

[85] Pedreiras P., Gai P., Almeida L., Buttazzo G.C. (2005). FTT-Ethernet: A flexible real-time communication protocol that supports dynamic QoS management on Ethernet-based systems. IEEE Trans. Ind. Inform..

[86] Corrêa T.P., Almeida L. (2017). Ultra short cycle protocol for partly decentralized control applications. Proceedings of the 2017 22nd IEEE International Conference on Emerging Technologies and Factory Automation (ETFA).

[87] Galli S., Scaglione A., Wang Z. (2011). For the grid and through the grid: The role of power line communications in the smart grid. Proc. IEEE.

[88] Kuzlu M., Pipattanasomporn M., Rahman S. (2015). Review of communication technologies for smart homes/building applications. Proceedings of the 2015 IEEE Innovative Smart Grid Technologies-Asia (ISGT ASIA).

[89] Sharma K., Saini L.M. (2017). Power-line communications for smart grid: Progress, challenges, opportunities and status. Renew. Sustain. Energy Rev..

[90] Texas Instruments Website RS-232, RS-422, RS-485 Serial Communication General Concepts. https://www.ni.com/white-paper/11390.

[91] Powell J., Eng P. Profibus and Modbus: A Comparison. https://www.scribd.com/document/340198066/Siemens-Profibus-and-Modbus-Comparison.

[92] Centenaro M., Vangelista L., Zanella A., Zorzi M. (2016). Long-range communications in unlicensed bands: The rising stars in the IoT and smart city scenarios. IEEE Wirel. Commun..

[93] Mahmood A., Javaid N., Razzaq S. (2015). A review of wireless communications for smart grid. Renew. Sustain. Energy Rev..

[94] Han D.M., Lim J.H. (2010). Design and implementation of smart home energy management systems based on zigbee. IEEE Trans. Consum. Electron..

[95] Alliance Z., Alliance H. Smart Energy Profile 2 Application Protocol Standard. https://zigbee.org/download/standard-smart-energy-profile-2-0/.

[96] Bisdikian C. (2001). An overview of the Bluetooth wireless technology. IEEE Commun. Mag..

[97] Jain R. (2014). Bluetooth and Bluetooth Smart.

[98] Gomez C., Oller J., Paradells J. (2012). Overview and Evaluation of Bluetooth Low Energy: An Emerging Low-Power Wireless Technology. Sensors.

[99] Riggio R., Rasheed T., Testi S., Granelli F., Chlamtac I. (2011). Interference and traffic aware channel assignment in WiFi-based wireless mesh networks. Ad Hoc Netw..

[100] Camps-Mur D., Garcia-Saavedra A., Serrano P. (2013). Device-to-device communications with Wi-Fi Direct: Overview and experimentation. IEEE Wirel. Commun..

[101] Ploennigs J., Ryssel U., Kabitzsch K. (2010). Performance analysis of the EnOcean wireless sensor network protocol. Proceedings of the 2010 IEEE Conference on Emerging Technologies and Factory Automation (ETFA).

[102] Newman H.M. (2013). BACnet: The Global Standard for Building Automation and Control Networks.

[103] Shelby Z., Bormann C. (2011). 6LoWPAN: The Wireless Embedded Internet.

[104] Yassein M.B., Mardini W., Khalil A. (2016). Smart homes automation using Z-wave protocol. Proceedings of the International Conference on Engineering & MIS (ICEMIS).

[105] Adelantado F., Vilajosana X., Tuset-Peiro P., Martinez B., Melia-Segui J., Watteyne T. (2017). Understanding the limits of LoRaWAN. IEEE Commun. Mag..

[106] Cho J., Jeong S., Kim Y. (2015). Commercial and research battery technologies for electrical energy storage applications. Prog. Energy Combust. Sci..

[107] Eddahech A., Briat O., Vinassa J.M. (2015). Performance comparison of four lithium–ion battery technologies under calendar aging. Energy.

[108] Sabihuddin S., Kiprakis A.E., Mueller M. (2014). A numerical and graphical review of energy storage technologies. Energies.

[109] Malhotra A., Battke B., Beuse M., Stephan A., Schmidt T. (2016). Use cases for stationary battery technologies: A review of the literature and existing projects. Renew. Sustain. Energy Rev..

[110] Battery University Website. https://www.batteryuniversity.com.

[111] World Economic Forum, 2012. The Top 10 Emerging Technologies for 2012. https://www.weforum.org/agenda/2012/02/the-2012-top-10-emerging-technologies/.

[112] Ku M.L., Li W., Chen Y., Liu K.R. (2016). Advances in Energy Harvesting Communications: Past, Present, and Future Challenges. IEEE Commun. Surv. Tutor..

[113] Mitcheson P.D., Yeatman E.M., Rao G.K., Holmes A.S., Green T.C. (2008). Energy harvesting from human and machine motion for wireless electronic devices. Proc. IEEE.

[114] Wan Z.G., Tan Y.K., Yuen C. (2011). Review on energy harvesting and energy management for sustainable wireless sensor networks. Proceedings of the 2011 IEEE 13th International Conference on Communication Technology (ICCT).

[115] Matiko J.W., Grabham N.J., Beeby S.P., Tudor M.J. (2014). Review of the application of energy harvesting in buildings. Meas. Sci. Technol..

[116] Zhou G., Huang L., Li W., Zhu Z. (2014). Harvesting ambient environmental energy for wireless sensor networks: A survey. J. Sens..

[117] Lee H.K.H., Wu J., Barbé J., Jain S.M., Wood S., Speller E.M., Li Z., Castro F.A., Durrant J.R., Tsoi W.C. (2018). Organic photovoltaic cells–promising indoor light harvesters for self-sustainable electronics. J. Mater. Chem. A.

[118] Eliades D.G., Michaelides M.P., Panayiotou C.G., Polycarpou M.M. (2013). Security-oriented sensor placement in intelligent buildings. Build. Environ..

[119] Yoganathan D., Kondepudi S., Kalluri B., Manthapuri S. (2018). Optimal sensor placement strategy for office buildings using clustering algorithms. Energy Build..

[120] Udwadia F.E. Optimal sensor location for geotechnical and structural identification. Proceedings of the 8th World Conference on Earthquake Engineering.

[121] Kammer D.C. (1991). Sensor placement for on-orbit modal identification and correlation of large space structures. J. Guid. Control. Dyn..

[122] Papadimitriou C., Beck J.L., Au S.K. (2000). Entropy-based optimal sensor location for structural model updating. J. Vib. Control..

[123] Seabrook T. (2016). Optimal Placement Strategies of Minimum Effective Sensors for Application in Smart Buildings. https://www.semanticscholar.org/paper/Optimal-Placement-Strategies-of-Minimum-Effective-Seabrook/.

[124] Akkaya K., Younis M. (2005). A survey on routing protocols for wireless sensor networks. Ad Hoc Netw..

[125] Warrier M.M., Kumar A. (2016). An energy efficient approach for routing in wireless sensor networks. Procedia Technol..

[126] Guleria K., Verma A.K. (2019). Comprehensive review for energy efficient hierarchical routing protocols on wireless sensor networks. Wirel. Netw..

[127] Bhattacharyya D., Kim T.H., Pal S. (2010). A comparative study of wireless sensor networks and their routing protocols. Sensors.

[128] Heinzelman W.B., Chandrakasan A.P., Balakrishnan H. (2002). An application-specific protocol architecture for wireless microsensor networks. IEEE Trans. Wirel. Commun..

[129] Lindsey S., Raghavendra C., Sivalingam K.M. (2002). Data gathering algorithms in sensor networks using energy metrics. IEEE Trans. Parallel Distrib. Syst..

[130] Manjeshwar A., Agrawal D. TEEN: A protocol for enhanced efficiency in WSNs. Proceedings of the First International Workshop on Parallel and Distributed Computing Issues in Wireless Networks and Mobile Computing.

[131] Manjeshwar A., Agrawal D.P. (2002). APTEEN: A hybrid protocol for efficient routing and comprehensive information retrieval in wireless sensor networks. Proceedings of the Symposium International Parallel and Distributed.

[132] Kulik J., Heinzelman W., Balakrishnan H. (2002). Negotiation-based Protocols for Disseminating Information in Wireless Sensor Networks. Wirel. Netw..

[133] Intanagonwiwat C., Govindan R., Estrin D. Directed Diffusion: A Scalable and Robust Communication Paradigm for Sensor Networks. Proceedings of the Sixth Annual International Conference on Mobile Computing and Networking (MOBICOM).

[134] Xu Y., Heidemann J., Estrin D. (2001). Geography-informed energy conservation for ad hoc routing. Proceedings of the 7th Annual International Conference on Mobile Computing and Networking.

[135] Yu Y. Geographical and Energy-Aware Routing: A Recursive Data Dissemination Protocol for Wireless Sensor Networ. https://pdfs.semanticscholar.org/11ca/e1f847d741052bffba9af8d9fbd39973fd94.pdf.

[136] Alasem R., Reda A., Mansour M. (2011). Location based energy-efficient reliable routing protocol for wireless sensor networks. Recent Researches in Communications, Automation, Signal. Processing, Nanotechnology, Astronomy and Nuclear Physics.

[137] Curry E., O’Donnel J., Corry E., Hasan S., Keane M., O’Riain S. (2013). Linking building data in the cloud: Integrating cross-domain building data using linked data. Adv. Eng. Inform..

[138] Gil D., Johnsson M., Mora H., Szymański J. (2019). Review of the Complexity of Managing Big Data of the Internet of Things. Complexity.

[139] Satyanarayanan M. (2017). The emergence of edge computing. Computer.

[140] Balaji B., Bhattacharya A., Fierro G., Gao J., Gluck J., Hong D., Johansen A., Koh J., Ploennigs J., Agarwal Y. (2018). Brick: Metadata schema for portable smart building applications. Appl. Energy.

[141] ASHRAE’s BACnet Committee, Project Haystack and Brick Schema Collaborating to Provide Unified Data Semantic Modeling Solution. https://www.ashrae.org/.

[142] Kayastha N., Niyato D., Hossain E., Han Z. (2014). Smart grid sensor data collection, communication, and networking: A tutorial. Wirel. Commun. Mob. Comput..

[143] Sorrell S. The Internet of Things: Consumer, Industrial & Public Services; Juniper Research, 2016. https://www.juniperresearch.com/researchstore/iot-m2m/internet-of-things.

[144] Manyika J., Chui M., Bisson P., Woetzel J., Dobbs R., Bughin J., Aharon D. Unlocking the Potential of the Internet of Things. https://www.mckinsey.com/business-functions/digital-mckinsey/.

[145] Habibzadeh M., Hassanalieragh M., Ishikawa A., Soyata T., Sharma G. (2017). Hybrid solar-wind energy harvesting for embedded applications: Supercapacitor-based system architectures and design tradeoffs. IEEE Circuits Syst. Mag..

[146] Bhuyan S., Hu J. (2013). A natural battery based on lake water and its soil bank. Energy.

[147] Trizcinski P., Nathan A., Karanassios V. (2017). Approaches to energy harvesting and energy scavenging for energy autonomous sensors and microinstruments. Proc. SPIE.

[148] Souza C.P., Carvalho F.B., Silva F.A., Andrade H.A., Silva N.D.V., Baiocchi O., Müller I. (2016). On harvesting energy from tree trunks for environmental monitoring. Int. J. Distrib. Sens. Netw..

[149] McGarry S., Knight C. (2011). The potential for harvesting energy from the movement of trees. Sensors.

[150] Tarelho J.P., dos Santos M.P.S., Ferreira J.A., Ramos A., Kopyl S., Kim S.O., Hong S., Kholkin A. (2018). Graphene-based materials and structures for energy harvesting with fluids—A review. Mater. Today.

